# Continuous external negative pressure improves oxygenation and respiratory mechanics in Experimental Lung Injury in Pigs – A pilot proof-of-concept trial

**DOI:** 10.1186/s40635-020-00315-1

**Published:** 2020-12-18

**Authors:** Martin Scharffenberg, Jakob Wittenstein, Moritz Herzog, Sebastian Tauer, Luigi Vivona, Raphael Theilen, Thomas Bluth, Thomas Kiss, Thea Koch, Giuseppe Fiorentino, Marcelo Gama de Abreu, Robert Huhle

**Affiliations:** 1 Pulmonary Engineering Group, Dept. of Anaesthesiology and Intensive Care Medicine, University Hospital Carl Gustav Carus at Technische Universität Dresden, Fetscherstrasse 74, 01307 Dresden, Germany; 2 Department of Pathophysiology and Transplantation, University of Milan, Via Francesco Sforza 35, 20122 Milano, Italia; 3 Devision of Respiratory Physiopathology, Monaldi Hospital, Naples, Italy

**Keywords:** Continuous external negative pressure, CENP, Negative pressure ventilation, Mechanical ventilation, Acute respiratory distress syndrome, ARDS, Lung mechanics, Pleural pressure, Transpulmonary pressure, Electrical impedance tomography

## Abstract

**Background:**

Continuous external negative pressure (CENP) during positive pressure ventilation can recruit dependent lung regions. We hypothesised that CENP applied regionally to the thorax or the abdomen only, increases the caudal end-expiratory transpulmonary pressure depending on positive end-expiratory pressure (PEEP) in lung-injured pigs. Eight pigs were anesthetised and mechanically ventilated in the supine position. Pressure sensors were placed in the left pleural space, and a lung injury was induced by saline lung lavages. A CENP shell was placed at the abdomen and thorax (randomised order), and animals were ventilated with PEEP 15, 7 and zero cmH_2_O (15 min each). On each PEEP level, CENP of − 40, − 30, − 20, − 10 and 0 cmH_2_O was applied (3 min each). Respiratory and haemodynamic variables were recorded. Electrical impedance tomography allowed assessment of centre of ventilation.

**Results:**

Compared to positive pressure ventilation alone, the caudal transpulmonary pressure was significantly increased by CENP of ≤ 20 cmH_2_O at all PEEP levels. CENP of – 20 cmH_2_O reduced the mean airway pressure at zero PEEP (*P* = 0.025). The driving pressure decreased at CENP of ≤ 10 at PEEP of 0 and 7 cmH_2_O (*P* < 0.001 each) but increased at CENP of – 30 cmH_2_O during the highest PEEP (*P* = 0.001). CENP of – 30 cmH_2_O reduced the mechanical power during zero PEEP (*P* < 0.001). Both elastance (*P* < 0.001) and resistance (*P* < 0.001) were decreased at CENP ≤ 30 at PEEP of 0 and 7 cmH_2_O. Oxygenation increased at CENP of ≤ 20 at PEEP of 0 and 7 cmH_2_O (*P* < 0.001 each). Applying external negative pressure significantly shifted the centre of aeration towards dorsal lung regions irrespectively of the PEEP level. Cardiac output decreased significantly at CENP -20 cmH_2_O at all PEEP levels (*P* < 0.001). Effects on caudal transpulmonary pressure, elastance and cardiac output were more pronounced when CENP was applied to the abdomen compared with the thorax.

**Conclusions:**

In this lung injury model in pigs, CENP increased the end-expiratory caudal transpulmonary pressure. This lead to a shift of lung aeration towards dependent zones as well as improved respiratory mechanics and oxygenation, especially when CENP was applied to the abdomen as compared to the thorax. CENP values ≤ 20 cmH_2_O impaired the haemodynamics.

## Background

In controlled mechanical ventilation, a positive pressure is applied to produce an inspiratory flow into the lungs (positive pressure ventilation, PPV). Although PPV may be lifesaving, it has the potential to damage the lungs, a phenomenon that has been termed ventilator-induced lung injury (VILI). PPV can cause over-distension of gravitationally non-dependent lung regions (volutrauma), as well as a cyclic collapse and reopening in gravitationally dependent regions (atelectrauma) [1]. During PPV, mechanical stress is transferred to the lungs and further amplified at the interface between opened and collapsed alveoli. These mechanisms are especially relevant in acute respiratory distress syndrome (ARDS), where lung aeration is usually inhomogeneous. Furthermore, PPV frequently induces circulatory depression due to increased intrathoracic pressure [2]. While healthy subjects may tolerate these changes [3, 4], they can be clinically relevant in patients with respiratory or haemodynamic impairment.

Negative pressure ventilation (NPV) mimics physiologic spontaneous breathing and is a possible alternative of conventional PPV. NPV applies alternating external negative pressure to move the chest wall and induces an intrathoracic negative pressure, leading to an inflow of air, and was shown to reduce atelectasis and to increase oxygenation compared with PPV [5, 6]. NPV with a continuous negative instead of alternating external pressure, in the following referred to as continuous external negative pressure (CENP), and as performed with a whole-body chamber, was shown to improve oxygenation in rabbits [7]. In piglets, CENP in addition to PPV was as effective in recruiting and stabilising alveoli as positive end-expiratory pressure (PEEP) [8]. CENP in addition to PPV was further shown to improve lung function, to reduce lung injury and to selectively recruit dependent lung areas in pigs [9, 10], as well as to improve lung function in ARDS patients [11]. In contrast to whole-body chambers, CENP can be obtained with a shell covering only the ventral thoracic-abdominal wall [12].

In this study, we aimed to determine whether CENP, as applied regionally on the thoracic or abdominal wall, and combined with PPV, improves the respiratory function and mechanics, as well as haemodynamics in a model of lung injury in pigs. We hypothesised that, depending on the level of PEEP, regional CENP would increase the end-expiratory transpulmonary pressure in dorsal-caudal lung regions (TP_caud_), leading to a redistribution of aeration and ventilation, as well as improved oxygenation and respiratory system mechanics.

## Methods

The protocol of the study was approved by the local animal welfare committee and the Government of the State of Saxony, Germany (DD24.1-5131/394/76; DD24.1-55131/394/8; TVV 5/201; NTP-ID: 00014251-1-0). All animals received the best care in compliance with the federal Principles of Laboratory Animal Care.

### Anaesthesia and instrumentation

Eight female pigs (57.96 ± 9.44 kg) were anaesthetised with intravenous (i.v.) midazolam (1 mg/kg/h) and ketamine (15 mg/kg/h, i.v.). The trachea was orally intubated (inner diameter 8.0, Rüsch, Germany), and animals were mechanically ventilated (Evita XL, Drägerwerk AG & Co. KGaA, Lübeck, Germany). Continuous muscle paralysis was achieved by atracurium (3 mg/kg/h, i.v.). During anaesthesia, a balanced crystalloid solution was infused continuously. Arterial blood pressure was maintained ≥ 60 mmHg by continuous i.v. infusion of norepinephrine as necessary. Surgical instrumentation included surgical preparation and catheterization of the right jugular vein and carotid artery, as well as the urinary bladder. A thermo-dilution pulmonary artery catheter was placed through an 8.5 Fr. central venous sheath. A gastric feeding tube as well as a commercially available oesophageal balloon catheter were introduced into the oesophagus, and the correct position of the latter was confirmed as described elsewhere [13].

### Mechanical ventilation

Initially, the lungs were ventilated with intermittent PPV (IPPV) with tidal volume (V_T_) of 6 mL/kg, the flow of 35 l/min, inspired fraction of oxygen (F_I_O_2_) of 1.0, and inspiratory to expiratory time ratio (I to E) of 1:1. Respiratory rate (RR) was set to achieve PaCO_2_ of 35 to 45 mmHg. PEEP was set to 5 cmH_2_O and airway pressure was limit of 45 cmH_2_O.

### Placement of pleural pressure sensors

Custom-made pleural pressure sensors were placed into the left pleural space by video-assisted thoracoscopy (VATS) with animals placed in the right lateral decubitus position. To allow surgical access, a bronchial blocker (Rüsch EZ Blocker, Teleflex, Wayne, PA, USA) was placed and one-lung ventilation conducted. During one-lung ventilation, V_T_ was set to 5 mL/kg and RR was adjusted to achieve PaCO_2_ of 35 to 45 mmHg. After the collapse of the left lung was achieved, three pressure sensors were placed as described recently [14]. Briefly, one sensor was placed each at the 4th to 5th rib ventral, 4th to 5th rib dorsal and the 8th to 9th rib dorsal. Two-lung ventilation was then re-established, and the lungs were re-expanded using continuous positive airway pressure (CPAP) of 40 cmH_2_O for 30 s.

### Induction of lung injury

Lung injury was induced by lung lavage with warmed (37 to 39 °C) 0.9 % saline (35 mL/kg). Four lavages were performed in the prone position; animals were turned to the supine position, and another four lavages were performed. During the induction of lung injury, the norepinephrine infusion was adjusted to keep mean arterial pressure > 60 mmHg, while the ventilator settings were kept unchanged. The procedure was stopped if PaO_2_/F_I_O_2_ < 100 mmHg for at least 30 min or the total number of lavages (*n* = 8) was achieved.

### Experimental protocol

**Fig. 1:**
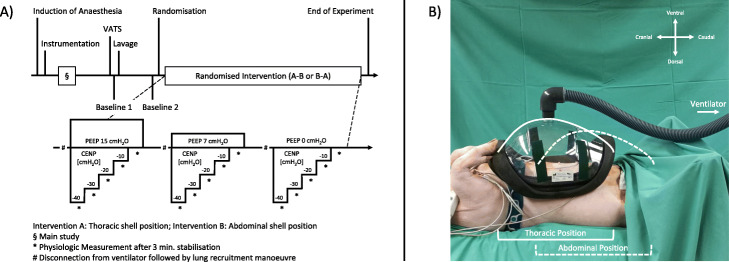
Time course of the experiment (**a**) and positioning of the shell on the pig thorax (**b**). *Legend:* VATS, video-assisted thoracoscopy; PEEP, positive end-expiratory pressure; CENP, continuous external negative pressure

The CENP shell was placed in thoracic or abdominal position, and the sequence of positions was randomised using sealed envelopes. In each position, PEEP levels of 15 cmH_2_O, 7 cmH_2_O and zero cmH_2_O were applied in this sequence (15 min each). Other ventilator settings were identical as previously described for two-lung ventilation. The lung volume history was reset prior each PEEP level by disconnection of the ventilator circuit, followed by a lung recruitment manoeuvre. The recruitment manoeuvre consisted of a stepwise increase of V_T_ at a constant PEEP of 15 cmH_2_O, until airway plateau pressure exceeded 40 cmH_2_O, as described elsewhere [4]. At each PEEP level, CENP of − 40, − 30, − 20, − 10 and 0 cmH_2_O was applied in this sequence (3 min each, Pegaso Vent, Dima, Italy). The sequence of interventions is illustrated in Fig. 1.

### Measurements

Respiratory as well as haemodynamic variables, including cardiac output (CO), were measured after 3 min of stabilisation at each CENP level. Maps of ventilation by electrical impedance tomography (EIT), flow, airway pressure and pleural pressures were recorded continuously, and tracings for the last 30 s of each step were used for this analysis.

#### Gas exchange and haemodynamics

Arterial blood gas analyses (aBGA) were performed just before starting each PEEP level as well as during CENP of − 40, − 20 and zero cmH_2_O using the ABL 80 Flex Basic (Radiometer, Denmark). Mean arterial (MAP), mean pulmonary artery pressures (MPAP), thermodilution CO and heart rate were obtained from the haemodynamic monitor (Philips IntelliVue MP70, Böblingen, Germany).

#### Respiratory signals and regional pleural pressure measurement

Airway pressure (P_AW_) and flow were acquired from the ventilator (Evita XL, Drägerwerk AG & Co. KGaA, Lübeck, Germany) using a custom-build software interacting with the Evita4Lab protocol. Regional pleural pressure at ventral, dorsal and caudal positions were measured continuously through custom-made air-tight chambers (30 × 30 × 1 mm each) connected via thin incompressible tubings to pressure transducers [14]. Furthermore, P_AW_ at the Y-piece and oesophageal pressure were measured continuously using pressure transducers (163PC01D48-PCB, FirstSensors AG, Berlin, Germany). The signals were analogue-to-digital converted and recorded for off-line analysis using a custom software (LabVIEW, National Instruments, Austin, TX, USA).

#### Respiratory system mechanics and mechanical power

Respiratory system elastance (E) and resistance (R) were determined by fitting of the equation of motion to the acquired respiratory signals by means of multiple linear regression. Additionally, the percentage of volume-dependent elastance was determined (%E_2_) [15, 16]. Regional transpulmonary pressures (TP) in the aforementioned regions were calculated by subtracting the corresponding pleural pressure from P_AW_. Mechanical work (MW) and energy (ME) were determined by numerical integration of the tidal pressure-volume curve by the trapezoidal rule [17]. Mechanical power per cycle (MP) was determined by multiplying ME and RR: MP = MW∙RR [18].

#### Distribution of aeration and ventilation

The distributions of aeration and ventilation were assessed by EIT (PulmoVista® 500, Drägerwerk AG & Co. KGaA, Lübeck, Germany) with an operating frequency of 130 kHz and 50 frames ∙ s^−1^. Raw measured EIT data were 50-Hz filtered and reconstructed using the manufacturer´s commercially available software. Reconstruction was done using the Dräger EIT Data Analysis Tool, Version 6.3. The reference slice for EIT reconstruction was manually set to correspond with the lung during the disconnected ventilator circuit just before the start of each PEEP level. Each EIT image of the resulting reconstructed temporal image series consisted of 32 × 32 pixels. The global region of interest (ROI) was defined a priori as a centred circle with radius of 16 pixel. This ROI was subdivided into four ROIs from ventral to dorsal, e.g. ventral, mid-ventral, mid-dorsal and dorsal. The centre of aeration (CoA) and the centre of ventilation (CoV) was determined as the position of the median impedance at end-expiration, and of the median impedance change, respectively, along the ventral-dorsal axis.

#### End of experiment

At the end of the protocol, pigs were killed under deep anaesthesia with 2 g thiopental i.v. followed by potassium chloride i.v. (50 mL, 1 M).

### Statistical analyses

Values are displayed as mean ± standard deviation (SD) or median [1^st^ to 3^rd^ quartile], as appropriate. Repeated measures ANOVA was applied to a linear mixed-effects model with factors position, PEEP and CENP [19]. Post hoc comparisons between CENP steps were adjusted according to Šidák [20]. Comparisons for each CENP step were performed using paired *t* test. Statistical significance was accepted at *P* < 0.05. All tests were conducted with the R statistical programming language [21].

## Results

All animals survived until the end of the protocol. In seven animals, injury was achieved with eight saline lavages, while one animal received seven lavages. The sequence of CENP shell positions did not affect the following results (*P* > 0.99).

### Regional pleural and transpulmonary pressures

**Fig. 2:**
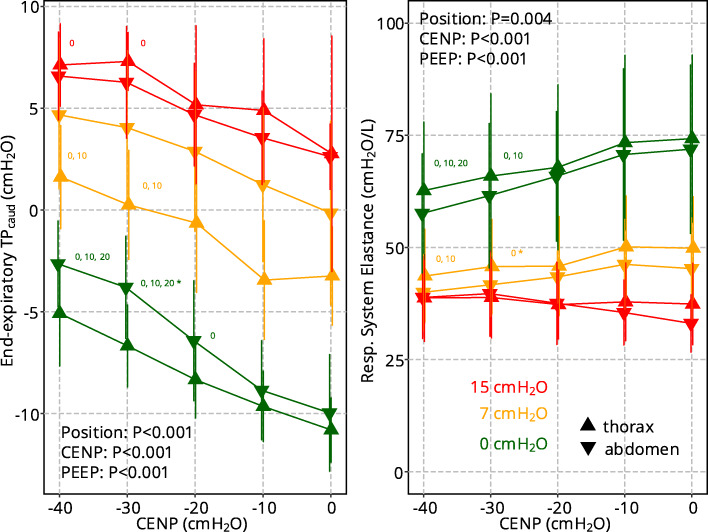
End-expiratory caudal transpulmonary pressure (left) and respiratory system elastance (right). *Legend:* CENP, continuous external negative pressure; PEEP, positive end-expiratory pressure. Triangles indicate different shell positions (abdomen and thorax), colours represent different PEEP levels, super-posed numbers indicate significance (*P* < 0.05) of the respective CENP level compared with the CENP level of the number and stars indicate difference between both positions

Both the shell position and the CENP levels significantly influenced pleural pressures (Table 1). At zero PEEP and CENP of – 30 cmH_2_O, end-expiratory TP_caud_ was significantly higher when the shell was placed in abdominal than in thoracic position (*P* < 0.001) (Fig. 2). At PEEP of 7 cmH_2_O, mean TP_caud_ became positive at CENP of – 10 cmH_2_O with the shell in abdominal position, but only at CENP of – 30 cmH_2_O in thoracic position. Depending on PEEP, lower CENP resulted in increased end-expiratory TP_caud_ (Table 1). Transpulmonary pressure gradient from ventral to caudal (TP_vent_-TP_caud_) decreased significantly with lowering CENP.

**Table 1:** Local pleural and corresponding transpulmonary pressures as measured by intra-pleural pressure sensors

Parameter	PEEP [cmH_2_O]	Position	CENP [cmH_2_O]					ANOVA (P-values)			
			0	-10	-20	-30	-40	Position	CENP	PEEP	Mixed
								<0.001	<0.001	<0.001	0.871
Ventral pleural pressure PP_vent_ (cmH_2_O)	0	abdomen	-2.89±6.2	-3.74±5.5	-5.54±4.7	-6.72±4.5	-7.19±4.4	0.051	<0.001		0.601
		thorax	-5.18±2.5	-5.24±2.9	-6.22±3.2	-7.05±3.2	-7.23±3.9				
						^0,10^	^0,10^				
	7	abdomen	-1.07±6.3	-1.44±5.7	-2.58±5	-3.48±4.5	-3.92±4.3	<0.001	0.099	^0^	0.968
		thorax	-4.1±3.6	-3.87±3.4	-4.75±3.6	-5.65±3.7	-5.67±4.4				
	15	abdomen	3.04±4.1	2.75±3.6	2.7±3.1	2.16±2.8	2.17±2.7	<0.001	0.933	^0,7^	0.990
		thorax	0.279±2.8	-0.355±2.7	-0.972±3	-1.12±3.6	-1.09±3.6				
					*		*				
Dorsal pleural pressure PP_dors_ (cmH_2_O)								0.183	<0.001	<0.001	0.248
	0	abdomen	4.39±6.2	3.04±5.6	1.48±5.1	-0.0648±4.6	-1.22±4.5	0.101	<0.001		0.989
		thorax	4.63±4.2	3.52±3.7	2.24±3.8	0.669±3.4	-0.505±3.3				
					^0^	^0,10^	^0,10,20^				
	7	abdomen	5.07±3.1	5.23±3	3.85±2.8	2.66±2.7	2.84±2.4	0.946	0.013	^0^	0.723
		thorax	5.03±4.3	4.11±4.4	4.1±3.2	3.28±3	3.27±3.2				
						^0^	^0^				
	15	abdomen	10.2±3.4	9.45±3.1	8.2±2.9	7.75±3	7.04±3	0.249	0.027	^0,7^	0.944
		thorax	11.3±3.8	10.2±3.4	9.06±3	7.79±2.6	7.11±2.5				
						^0^	^0,10^				
Caudal pleural pressure PP_caud_ (cmH_2_O)								<0.001	<0.001	<0.001	0.580
	0	abdomen	11.1±2.5	9.89±2.3	7.69±2.8	5.18±2.3	4.1±2.6	0.006	<0.001		0.590
		thorax	11.5±1.7	10.2±1.6	9.06±2	7.43±2	5.85±2.5				
					^0^	^0,10,20^	^0,10,20^				
	7	abdomen	7.99±4.6	7.2±4	5.98±4.2	4.95±4.1	4.34±3.9	<0.001	0.027		0.930
		thorax	11.4±2.4	11.1±2.3	8.85±2.9	7.98±2.4	6.62±2.2				
				*		^0,10^	^0,10^				
	15	abdomen	13.7±1.6	12.7±2.4	11.8±2.7	10.6±2.7	10.2±2.7	0.323	0.025	^0,7^	0.947
		thorax	13.9±6.1	11.8±4.2	11.5±4.4	9.47±2.5	9.66±2.5				
						^0^	^0^				
Ventral trans-pulmonary pressure TP_vent_ (cmH_2_O)								<0.001	<0.001	<0.001	0.771
	0	abdomen	4.02±5.9	4.78±5.1	6.81±4.4	7.81±4.2	8.65±4.1	0.330	<0.001		0.524
		thorax	5.89±2.3	5.83±2.6	6.95±3.1	7.8±3	8±3.8				
						^0.10^	^0,10^				
	7	abdomen	8.15±6.6	9.89±6.1	11.4±5.5	12.5±5.2	12.9±5	<0.001	0.002	^0^	0.609
		thorax	12.3±3.6	12±3.3	13±3.6	13.9±3.4	15.2±3.9				
						^0^	^0.10^				
	15	abdomen	13.2±4.2	13.5±3.8	13.8±3.1	14.7±2	14.6±2.1	<0.001	0.557	^0,7^	0.989
		thorax	16.3±1.7	17±1.5	17.7±2	17.9±2.9	17.9±3.1				
				*	*	*	*				
Dorsal trans-pulmonary pressure TP_dors_ (cmH_2_O)								0.009	<0.001	<0.001	0.600
	0	abdomen	-3.26±6	-2.01±5.3	-0.207±4.6	2.69±4.6	2.68±3.9	<0.001	<0.001		0.630
		thorax	-3.92±3.9	-2.94±3.4	-1.52±3.4	0.0791±3	1.28±2.9				
					^0^	^0,10,20^ *	^0,10,20^				
	7	abdomen	2.43±3.1	3.22±2.5	5.01±2.2	6.35±2.6	6.46±2.3	0.141	<0.001	^0^	0.529
		thorax	3.15±4.2	2.85±3.5	4.1±2.9	4.95±2.6	5.33±2.7				
						^0,10^	^0,10^				
	15	abdomen	6.09±3.1	6.82±3.1	8.33±2.9	9.14±3.7	9.7±3.3	0.399	0.002	^0,7^	0.986
		thorax	5.35±3.7	6.43±3.2	7.65±2.7	8.99±2.5	9.67±2.7				
					^0^	^0,10^	^0,10^				
TP_vent_ – TP_caud_ (cmH_2_O)								<0.001	0.003	<0.001	0.997
	0	abdomen	14±7.5	13.6±6.4	13.2±5.8	11.4±4.5	11.3±4.4	0.001	0.031		0.970
		thorax	16.7±2.8	15.5±3.5	15.3±3	14.5±2.9	13.1±2.7				
	7	abdomen	9.26±5.3	8.64±4.3	8.56±4.6	8.43±4.4	7.93±4.8	<0.001	0.484	^0^	0.913
		thorax	15.5±3.1	15.3±2.6	13.6±4.1	13.6±3.9	12.6±4				
				*							
	15	abdomen	10.6±4.2	9.96±3.7	9.14±3.8	8.45±3.3	7.98±3.1	<0.001	0.063	^0, 7^	0.978
		thorax	13.6±4.8	12.1±2.4	12.5±2.4	10.6±2.6	10.7±3.3				
							*				

Values are mean ± standard deviation. Linear-mixed effects, repeated-measures ANOVA with factors position (abdomen or thorax), PEEP and CENP were performed for each parameter (1^st^ line) over all values and for each PEEP-step (1^st^ line of each PEEP step). Post hoc comparison was done acc. to Sidak after significant effects for CENP (0, 10, 20 and 30 indicate if the value at respective CENP was different compared to CENP 0, 10, 20 and 30 cmH_2_O, respectively.) or PEEP (0 or 7 in PEEP effect column indicates the significant difference between the respective PEEP row with PEEP level indicated in the number, e.g. ^0^ or ^7^) or position (* position effects at the respective CENP). Significance was accepted for *P* < 0.05

*CENP* continuous external negative pressure, *PEEP* positive end-expiratory pressure

### Other respiratory variables

Respiratory variables are summarised in Table 2. V_T_ and RR did differ neither between CENP levels nor between shell positions. Minute ventilation was significantly lower with the shell in thoracic position at PEEP of both zero and 15 cmH_2_O. Peak P_AW_ was reduced at CENP ≤ 20 cmH_2_O at PEEP of 0 and 7 cmH_2_O, but not affected by shell position. The effects of the shell position on driving pressure (∆P_AW_) did not reach statistical significance in post hoc tests. However, the driving pressure decreased at CENP of ≤ 10 with PEEP of 0 and 7 cmH_2_O (*P* < 0.001 each), but increased at CENP of – 30 cmH_2_O during highest PEEP (*P* = 0.001). Compared to zero CENP, the applied MP was significantly lower at CENP levels lower than – 30 cmH_2_O and zero PEEP (Table 2).

**Table 2:** Ventilator settings, airway pressure variables and mechanical power

Parameter	PEEP [cmH_2_O]	Position	CENP [cmH_2_O]					ANOVA (P-values)			
			0	-10	-20	-30	-40	Position	CENP	PEEP	Mixed
Tidal volume V_T_ (mL/kg)								0.801	0.081	0.694	0.997
	0	abdomen	5.77±0.4	5.71±0.4	5.67±0.3	5.65±0.4	5.76±0.4	0.070	0.708		0.620
		thorax	5.79±0.5	5.71±0.4	5.83±0.5	5.83±0.5	5.81±0.5				
	7	abdomen	5.8±0.5	5.71±0.4	5.78±0.4	5.89±0.4	5.83±0.4	0.109	0.694		0.723
		thorax	5.78±0.4	5.58±0.4	5.72±0.3	5.67±0.4	5.82±0.5				
	15	abdomen	5.78±0.4	5.67±0.3	5.72±0.4	5.72±0.4	5.8±0.4	0.713	0.689		0.922
		thorax	5.73±0.4	5.65±0.5	5.77±0.4	5.74±0.5	5.72±0.5				
Respiratory rate RR (min^-1^)								0.540	0.088	0.005	0.403
	0	abdomen	21±7.6	21.4±8.5	21.2±7.6	20.9±7.6	21±7.8	0.064	0.933		0.978
		thorax	20.6±7.7	21±9.1	20.5±8.4	20.5±8	20.1±7.9				
	7	abdomen	17.3±4	20.8±9.2	20.3±7.4	20.1±7.1	20.9±8.2	0.639	0.166	^0^	0.536
		thorax	19.6±7.2	22.2±8.9	19.8±7.4	19.9±7.3	19.6±7.7				
	15	abdomen	19.8±7	20.1±7.4	19.9±7.2	19.9±7	19.8±7.5	0.110	0.956	^0,7^	0.983
		thorax	19.5±7.1	19.9±7.8	19.5±7.5	19.4±7.1	19.6±7.7				
Minute volume ventilation MV (L)								0.030	0.735	<0.001	0.517
	0	abdomen	6.8±2.1	6.8±2.1	6.7±2.0	6.8±2100	6.7±1.9	0.045	0.968		0.997
		thorax	6.6±2.1	6.6±2.1	6.6±2.1	6.6±2100	6.4±2.0				
	7	abdomen	5.6±1.2	6.6±2.2	6.6±2.0	6.6±2.0	6.6±2.1	0.565	0.051	^0^	0.315
		thorax	6.3±1.7	6.2±1.7	6.3±1.7	6.2±1.7	6.4±1.7				
	15	abdomen	6.4±2.2	6.4±2.2	6.4±2.1	6.4±2.1	6.4±2.1	0.001	0.997	^0,7^	0.996
		thorax	6.2±1.8	6.2±1.7	6.2±1.8	6.1±1.7	6.2±1.7				
Mean airway pressure mean P_AW_ (cmH_2_O)								0.817	0.025	<0.001	0.009
	0	abdomen	11.9±2	11.3±2.1	10.7±2	10.7±1.7	9.84±2.1	0.534	<0.001		0.780
		thorax	11.6±1.7	11.4±1.8	11±1.9	10.7±1.9	10.2±1.8				
					^0^	^0^	^0,10,20^				
	7	abdomen	15.2±1	15.3±1.7	14.9±1.6	14.8±1.6	14.8±1.5	0.471	0.550	^0^	0.552
		thorax	15.5±1.4	14.8±1.2	14.8±1.3	14.6±1.3	14.6±1.4				
	15	abdomen	21.4±1.4	21.5±1.5	21.7±1.5	22±1.5	22±1.4	0.696	0.792	^0,7^	0.983
		thorax	21.8±1.6	21.7±1.4	21.8±1.4	21.9±1.4	21.9±1.4				
Peak airway pressure peak P_AW_ [cmH_2_O]								0.821	<0.001	<0.001	<0.001
	0	abdomen	29.5±3.6	28.4±3.7	26.8±3.7	26.8±2.7	24.7±3.9	0.214	<0.001		0.766
		thorax	29.3±3.5	28.7±3.5	27.7±3.7	26.9±3.9	25.8±3.9				
					^0^	^0,10^	^0,10,20^				
	7	abdomen	28.5±2.1	28±2.5	27.3±2.5	27.1±2.4	26.9±2	0.572	0.023		0.797
		thorax	28.9±2.8	27.5±2.2	27.3±2.5	26.9±2.5	26.6±2.9				
					^0^	^0^	^0^				
	15	abdomen	31.8±2	31.9±2.1	32.7±2.1	33.3±2.1	33.3±1.9	0.636	0.098	^0,7^	0.873
		thorax	32.2±2.1	32±2.1	32.4±2.1	32.8±2.3	32.7±2.3				
Airway driving pressure ∆P_AW_ (cmH_2_O)								0.045	<0.001	<0.001	<0.001
	0	abdomen	24.5±3.2	23.8±3.3	22.3±3.4	22.5±2.8	20.4±3.6	0.088	<0.001		0.635
		thorax	24.3±3.5	24.4±3.4	23.3±3.9	22.7±4	21.7±4				
					^0^	^0^	^0,10,20^				
	7	abdomen	17.8±2.4	17±2.5	16.2±2.2	16±1.9	15.5±1.9	0.227	<0.001	^0^	0.761
		thorax	18.2±2.8	16.8±2.1	16.7±2.5	16.2±2.7	16.2±3.2				
				^0^	^0^	^0^	^0,10^				
	15	abdomen	12.7±1.4	13±1.3	13.6±1.4	14.4±1.1	14.2±1.1	0.288	0.001	^0,7^	0.425
		thorax	13.6±2.4	13.5±2.5	13.7±2.3	14.1±2.8	14.1±2.7				
						^0^					
Positive end-expiratory pressure PEEP (cmH_2_O)								0.411	0.894	<0.001	0.936
	0	abdomen	0.313±1.2	0.209±1.2	0.172±1.2	0.341±1.4	0.208±1.2	0.177	0.885		0.947
		thorax	0.146±0.67	0.129±0.68	0.11±0.67	0.104±0.69	0.17±0.66				
	7	abdomen	6.62±0.66	7.05±1.5	7.18±1.4	7.23±1.3	7.3±1.2	0.343	0.015	^0^	0.399
		thorax	6.86±1	7.03±0.85	6.88±0.99	6.96±1	7.19±0.93				
							^0^				
	15	abdomen	15.3±1.3	15.4±1.3	15.4±1.3	15.3±1.3	15.4±1.3	0.908	1.000	^0,7^	1.000
		thorax	15.4±1.7	15.3±1.5	15.3±1.4	15.3±1.4	15.3±1.3				
Mechanical power MP (J/min)								0.687	0.009	<0.001	0.010
	0	abdomen	12.7±4.8	12±4.4	11±3.9	10.5±3.5	9.9±3.4	0.329	<0.001		0.805
		thorax	12.4±4.8	12±4.2	11.6±4.2	11.2±3.9	10.3±3.2				
						^0^	^0,10^				
	7	abdomen	7.4±1.5	8.08±2.9	7.36±1.8	7.27±1.7	7.36±2	0.957	0.448	^0^	0.587
		thorax	8.1±2.2	7.81±1.9	7.34±1.9	7.04±1.8	7.12±2				
	15	abdomen	5.97±2.4	6.02±2.5	6.17±2.5	6.38±2.7	6.41±2.6	0.004	0.663	^0,7^	0.947
		thorax	5.69±1.4	5.6±1.3	5.77±1.4	5.75±1.2	5.76±1.3				
Index of over-distension %E_2_ (%)								0.787	0.262	<0.001	0.535
	0	abdomen	-28.5±5.3	-27.9±5.1	-27.5±3.1	-25.4±2.4	-24.1±2.9	<0.001	0.025		0.565
		thorax	-30.7±7.8	-30.4±6.9	-30.7±5.5	-30.3±4.5	-29.2±4.7				
							*				
	7	abdomen	0.00224±7	1.69±7.8	2.49±8.7	7.24±10	5.78±8.3	0.011	0.404	^0^	0.745
		thorax	-8.1±12	-5.78±12	-1.84±13	4.29±13	4.58±19				
				*							
	15	abdomen	49.8±16	42.4±17	44.3±14	42.8±13	43.5±14	0.002	0.632	^0,7^	0.505
		thorax	48.6±23	52±23	53.2±22	53.8±27	51.5±26				
Resistance R (cmH_2_O·s/l)								0.674	<0.001	<0.001	0.015
	0	abdomen	0.244±0.043	0.235±0.043	0.218±0.034	0.209±0.027	0.191±0.033	0.014	<0.001		0.759
		thorax	0.247±0.043	0.242±0.037	0.234±0.034	0.224±0.028	0.215±0.027				
						^0^	^0,10^				
	7	abdomen	0.177±0.028	0.171±0.027	0.158±0.027	0.157±0.03	0.154±0.026	0.546	0.005	^0^	0.948
		thorax	0.172±0.015	0.167±0.014	0.161±0.018	0.154±0.019	0.153±0.02				
						^0^	^0,10^				
	15	abdomen	0.138±0.037	0.136±0.032	0.135±0.032	0.133±0.024	0.138±0.021	<0.001	0.956	^0,7^	0.996
		thorax	0.121±0.019	0.121±0.023	0.122±0.02	0.12±0.023	0.122±0.026				

Values are mean ± standard deviation. Linear-mixed effects, repeated-measures ANOVA with factors position (abdomen or thorax), PEEP and CENP were performed for each parameter (1^st^ line) over all values and for each PEEP-step (1^st^ line of each PEEP step). Post hoc comparison was done according to Sidak after significant effects for CENP (0, 10, 20 and 30 indicate if the value at respective CENP was different compared to CENP 0, 10, 20 and 30 cmH_2_O, respectively) or PEEP (0 or 7 in PEEP effects column indicate the significant difference between the respective PEEP row with PEEP level indicated in the number, e.g. 0 or 7) or Position (* position effects at the respective CENP). Significance was accepted for *P* < 0.05

*CENP* continuous external negative pressure, *PEEP* positive end-expiratory pressure

The respiratory system mechanics are described in Table 3. The elastance was lower with the shell in abdominal than in thoracic position at CENP of – 30 cmH_2_O when combined with PEEP of 7 cmH_2_O. CENP ≤ 30 cmH_2_O significantly decreased E at PEEP of 0 and 7 cmH_2_O (Fig. 2), but post hoc tests did not reveal differences at PEEP of 15 cmH_2_O. While the resistance was not significantly affected by the shell position, CENP ≤ 30 cmH_2_O decreased R at PEEP of 0 and 7 cmH_2_O but not at PEEP of 15 cmH_2_O. The index of over-distension (%E_2_) was not affected by the shell position or CENP level (Table 2).

**Table 3:** Gas exchange and haemodynamics

Parameter	PEEP (cmH_2_O)	Position	CENP (cmH_2_O)					ANOVA (P-values)			
			0	− 10	− 20	− 30	− 40	Position	CENP	PEEP	Mixed
Mean arterial pressure, MAP (mmHg)								0.080	< 0.001	< 0.001	< 0.001
	0	Abdomen	93 ± 22	89.6 ± 21	89.1 ± 20	88.4 ± 13	91.2 ± 9	0.271	0.899		0.996
		Thorax	93.9 ± 24	92.6 ± 20	91 ± 18	91.9 ± 11	94.5 ± 11				
	7	Abdomen	96.5 ± 10	94.5 ± 13	89.5 ± 11	87.2 ± 8.7	83.8 ± 10	0.349	0.006		0.137
		Thorax	91.8 ± 11	92 ± 9.6	92 ± 7.9	93.1 ± 7.9	90.8 ± 5.4				
	15	Abdomen	97.1 ± 13	88.6 ± 15	77.2 ± 9.3	70.4 ± 9.8	62.8 ± 13	0.107	< 0.001	^0, 7^	0.095
		Thorax	90.1 ± 11	88.5 ± 11	85.8 ± 12	78.2 ± 11	71.4 ± 14				
					^0^	^0,10^	^0,10,20^				
Heart rate, HR (bpm)								0.135	0.959	0.005	0.114
	0	Abdomen	90 ± 28	88.6 ± 27	88.1 ± 27	86.9 ± 26	85.5 ± 23	0.141	0.716		0.734
		Thorax	90.9 ± 28	88.2 ± 27	85 ± 27	83.4 ± 27	80.6 ± 24				
	7	Abdomen	84 ± 22	82.6 ± 22	83.5 ± 21	83.9 ± 20	86.5 ± 20	0.022	0.841	^0^	0.989
		Thorax	81 ± 22	79.5 ± 21	80.5 ± 22	80.6 ± 22	81.2 ± 22				
	15	Abdomen	75.1 ± 32	89.6 ± 21	92.4 ± 23	91.6 ± 23	91.4 ± 25	0.830	0.092		0.530
		Thorax	85.9 ± 21	86.1 ± 19	87.4 ± 20	88 ± 21	89.2 ± 21				
								< 0.001	< 0.001	< 0.001	0.278
Mean pulmonary airway pressure, MPAP (mmHg)	0	Abdomen	28.8 ± 6.3	26.5 ± 5.7	24 ± 6	22 ± 6.2	20.4 ± 5	< 0.001	< 0.001		0.168
		Thorax	29 ± 5.1	28.8 ± 5.1	27.2 ± 5.6	25.8 ± 5.5	28.1 ± 15				
					*	^0^*	^0^				
	7	Abdomen	25.1 ± 3.2	23.1 ± 4.2	19.4 ± 3.6	18.5 ± 3.6	18 ± 3.5	< 0.001	< 0.001	^0^	0.042
		Thorax	25.8 ± 4.3	24.5 ± 4.7	23 ± 4.6	21.8 ± 4.8	21.1 ± 4.2				
					^0, 10^*	^0, 10^*	^0, 10^*				
	15	Abdomen	21.5 ± 2.9	20.4 ± 2.8	19.2 ± 3.1	17.4 ± 5.5	19.2 ± 3.7	< 0.001	< 0.001	^0, 7^	0.129
		Thorax	22.4 ± 4.8	21.5 ± 4.5	20.8 ± 3.9	21.2 ± 4.8	21.8 ± 4.7				
					^0^	^0^*	*				
Infusion rate of noradrenaline (mg/h)								0.026	0.970	0.038	0.973
	0	Abdomen	0.28 ± 0.6	0.28 ± 0.6	0.18 ± 0.3	0.18 ± 0.3	0.18 ± 0.3	0.008	0.328		0.642
		Thorax	0.13 ± 0.2	0.13 ± 0.2	0.13 ± 0.2	0.14 ± 0.2	0.14 ± 0.2				
	7	Abdomen	0.07 ± 0.1	0.07 ± 0.1	0.07 ± 0.1	0.08 ± 0.1	0.08 ± 0.1	0.013	1.000		0.978
		Thorax	0.13 ± 0.2	0.13 ± 0.2	0.14 ± 0.2	0.14 ± 0.2	0.19 ± 0.4				
	15	Abdomen	0.07 ± 0.1	0.08 ± 0.1	0.10 ± 0.1	0.12 ± 0.2	0.12 ± 0.2	0.007	0.999		0.988
		Thorax	0.25 ± 0.5	0.31 ± 0.6	0.36 ± 0.8	0.41 ± 0.9	0.46 ± 1				
pH (arb. un.)								< 0.001	0.116	< 0.001	0.234
	0	Abdomen	7.39 ± 0.06	n/a	7.40 ± 0.07	n/a	7.41 ± 0.06	0.054	0.040		0.878
		Thorax	7.38 ± 0.07	n/a	7.38 ± 0.08	n/a	7.40 ± 0.07				
	7	Abdomen	7.42 ± 0.06	n/a	7.44 ± 0.07	n/a	7.43 ± 0.07	0.004	0.211	^0^	0.413
		Thorax	7.42 ± 0.07	n/a	7.42 ± 0.08	n/a	7.41 ± 0.06				
							*				
	15	Abdomen	7.44 ± 0.06	n/a	7.44 ± 0.069	n/a	7.44 ± 0.063	0.022	0.996	^0, 7^	0.738
		Thorax	7.42 ± 0.08	n/a	7.43 ± 0.071	n/a	7.42 ± 0.081				
Arterial partial pressure of CO_2_, PaCO_2_ (mmHg)								0.054	0.152	< 0.001	0.107
	0	Abdomen	42.4 ± 4.2	n/a	41.3 ± 7.3	n/a	40.1 ± 8.7	0.044	0.666		0.456
		Thorax	47.8 ± 16.0	n/a	43.7 ± 9.0	n/a	41.2 ± 10				
	7	Abdomen	38.1 ± 7.4	n/a	38.2 ± 7.1	n/a	36.8 ± 8.8	0.685	0.462	^0^	0.640
		Thorax	37.8 ± 8.5	n/a	38.3 ± 9.4	n/a	38.1 ± 9.2				
	15	Abdomen	35.7 ± 6.9	n/a	35.3 ± 7.7	n/a	36.8 ± 9.1	0.525	0.580	^0, 7^	0.804
		Thorax	37.0 ± 9.7	n/a	35.9 ± 9.1	n/a	36.6 ± 10				

Values are mean ± standard deviation. Linear-mixed effects, repeated-measures ANOVA with factors position (abdomen or thorax), PEEP and CENP were performed for each parameter (1^st^ line) over all values and for each PEEP-step (1^st^ line of each PEEP step). Post hoc comparison was done according to Sidak after significant effects for CENP (0, 10, 20 and 30 indicate if the value at respective CENP was different compared to CENP 0, 10, 20 and 30 cmH_2_O, respectively) or PEEP (0 or 7 in PEEP effects column indicate the significant difference between the respective PEEP row with PEEP level indicated in the number, e.g. ^0^ or ^7^) or position (* position effects at the respective CENP). Significance was accepted for *P*< 0.05

*CENP* continuous external negative pressure, *PEEP* positive end-expiratory pressure, *n/a* not available

### Gas exchange

**Fig. 3:**
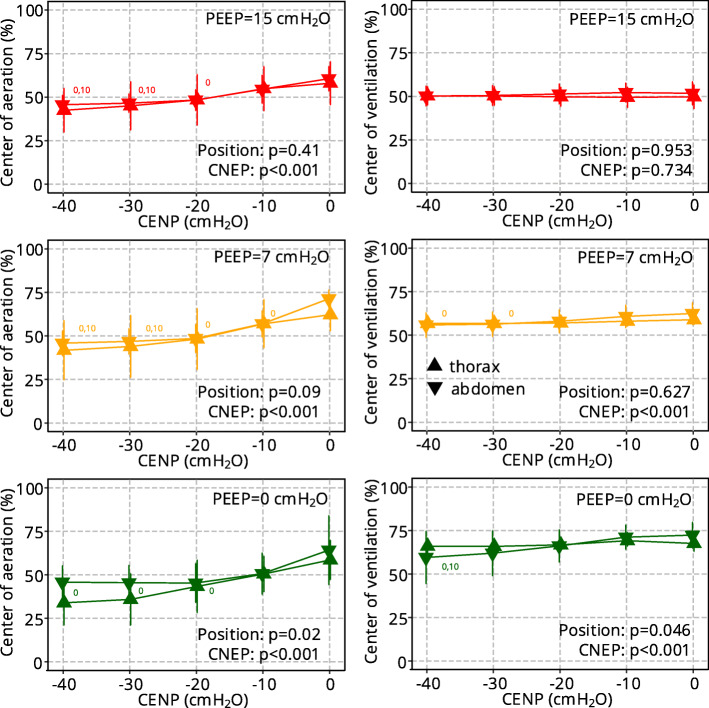
Arterial oxygenation (PaO_2_, left) and cardiac output (CO, right). *Legend:* CENP, continuous external negative pressure; PEEP, positive end-expiratory pressure. Triangles indicate different shell positions (abdomen and thorax), colours represent different PEEP levels, super-posed numbers indicate significance (*P* < 0.05) of the respective CENP level compared with the CENP level of the number and stars indicate difference between both positions

Following lung lavage, PaO_2_/F_I_O_2_ dropped from 672.63 ± 57.27 to 57.74 ± 19.58 mmHg (*P* < 0.001). At PEEP of 7 cmH_2_O and CENP of – 20 cmH_2_O, PaO_2_/F_I_O_2_ was significantly higher with the shell in abdominal than in thoracic position (Fig. 3). CENP of - 40 and – 20 cmH_2_O significantly increased PaO_2_/F_I_O_2_ at PEEP of 0 and 7 cmH_2_O, respectively, but not at PEEP of 15 cmH_2_O. Further variables of gas exchange during interventions are described in Table 3. PaCO_2_ and arterial pH did not differ between shell positions or CENP levels. Accordingly, arterial pH was similar between CENP levels.

### Distribution of aeration and ventilation

CENP shifted the CoA and CoV towards dependent (dorsal) lung regions at every PEEP level (Fig. 3). While a CENP of – 10 cmH_2_O induced significant changes at PEEP of 7 cmH_2_O, a CENP of – 20 cmH_2_O redistributed aeration at PEEP of 15 cmH_2_O.

### Haemodynamics

**Fig. 4:**
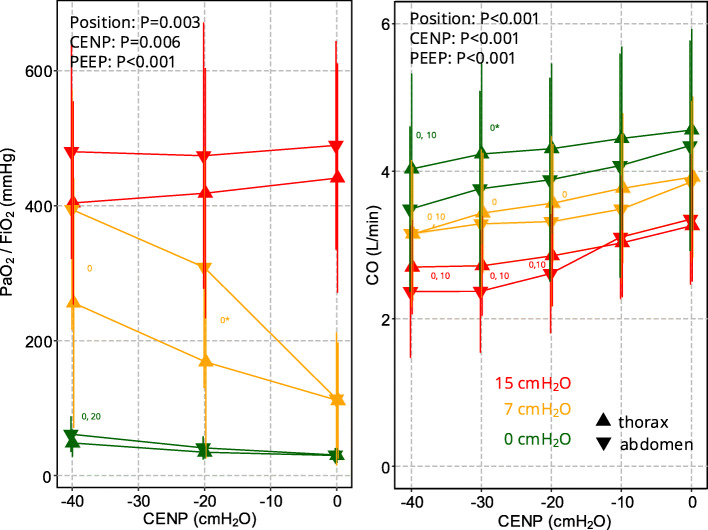
Centre of aeration (left) and centre of ventilation (right) as a percentage from dorsal. *Legend:* CENP, continuous external negative pressure; PEEP, positive end-expiratory pressure. Triangles indicate different shell positions (abdomen and thorax), colours represent different PEEP levels, super-posed numbers indicate significance (*P* < 0.05) of the respective CENP level compared with the CENP level of the number and stars indicate the difference between both positions

CO and MPAP were lower during abdominal compared to thoracic CENP at zero PEEP and CENP of – 30 cmH_2_O (Fig. 4, Table 3). At zero PEEP, CO decreased with both lowest CENP and highest PEEP levels. MAP was not affected by shell position, but decreased significantly at CENP ≤ 20 cmH_2_O and PEEP of 15 cmH_2_O.

## Discussion

We found that in this model of acute lung injury in pigs under PPV, CENP (1) increased transpulmonary pressures in dorsal lung regions, (2) shifted aeration and ventilation towards dependent zones, (3) increased oxygenation, (4) reduced driving pressure and mechanical power and (5) decreased mean arterial pressure and cardiac output. These effects were dependent on PEEP, but observed mainly with CENP ≤ 20 cmH_2_O and applied to the abdomen.

To the best of our knowledge, this was the first study examining the effects of regionally applied CENP on respiratory function and mechanics in experimental lung injury. Our study has strengths. Both the animal species and the lung injury model represent established methods of experimental investigations on mechanical ventilation and VILI [22]. The saline lung lavage induced a significant impairment of oxygenation, which met the severe ARDS criteria according to the Berlin definition [23]. Of note, a lavage-only model cannot replicate all characteristics of clinical presentation of ARDS. However, this was not necessary herein as we sought to use a model of recruitable atelectasis to investigate the effects of CENP levels and positions and did not analyse pulmonary inflammation [24]. The appropriateness of the model was underlined by significant differences in the distribution of ventilation and oxygenation, which depended upon the shell position, as well as CENP and PEEP levels.

Confirming our main hypothesis, CENP resulted in higher end-expiratory TP_caud_. This finding is in line with another study showing that CENP applied to the thorax and abdomen increased transpulmonary pressures [10]. However, in our study, such effects were achieved with the regional application of CENP instead of a whole-body chamber. In our trial, TP_caud_ obtained with PEEP of 7 cmH_2_O and CENP of – 20 cmH_2_O was similar to that achieved with PEEP of 15 cmH_2_O without CENP. Accordingly, our results are in line also with another study showing that improved lung recruitment was achieved at lower distending pressures when external negative pressure was applied to PPV with PEEP [25]. Our EIT analyses revealed namely that the ventilation of dorsal zones increased when CENP was applied. In fact, the distribution of ventilation and TP_caud_ obtained with PEEP of 7 cmH_2_O combined with CENP of – 20 cmH_2_O were similar to that observed with PEEP of 15 cmH_2_O without CENP. Differences between PPV alone and PPV + CENP may be explained by the non-selective increase of the global transpulmonary pressure and consecutive persistence of the physiologic dorso-ventral pleural pressure gradient during PPV with PEEP. In fact, the application of external negative pressure on the abdomen CENP decreased the ventro-dorsal pleural pressure gradient in another experimental study [25]. The vertical gradient of transpulmonary pressure, which is associated with lung collapse in dependent lung regions, is induced by gravitational forces [26]. One hypothesis of applying local negative pressure externally to the thorax is to decrease or even invert this vertical gradient to reduce derecruitment and shift the ventilation towards dependent lung regions. Indeed, the transpulmonary pressure gradient from ventral to caudal regions decreased with lowering CENP.

It is worth noting that CENP more effectively increased TP_caud_ when CENP was applied to the abdomen than to the thorax. A possible explanation is that the shape and stiffness of the porcine thorax differs from that of humans. Also, abdominal CENP may more effectively dislocate the diaphragm [10], resulting in more pronounced effects on pleural pressures. The dislocation of the diaphragm increased end-expiratory lung volume (EELV) in a bacterial infection model in sheep [26]. Although EELV was not assessed in our study, the results of the gas exchange suggest an increase EELV comparable to that resulting from PEEP alone.

CENP increased end-expiratory TP_caud_, which reached positive values at certain combinations of CENP and PEEP levels. Indeed, at PEEP of 7 cmH_2_O, TP_caud_ was significantly higher with CENP of – 20 cmH_2_O than without CENP. Accordingly, the CoA was significantly shifted towards dorsal regions and oxygenation was significantly higher with these settings. These results indicate effective recruitment induced by CENP on moderate PEEP and are in line with other experimental studies [9, 10]. Although TP_caud_ increased and ventilation was shifted towards dependent lung zones with CENP, oxygenation improved only at the lowest level of CENP during zero PEEP and did not improve further during the highest PEEP. Possibly, the recruitment of lungs achieved a maximum at those CENP and PEEP levels. Our observation that CENP did not improve the respiratory system elastance but driving pressure increased at a PEEP of 15 cmH_2_O, further supports this hypothesis. In fact, this behaviour resembles the sigmoidal relationship between lung volume and airway pressure. Worthwhile noting, for a high PEEP of 15 cmH_2_O, a value well above PEEP corresponding to best compliance (values not shown), both transpulmonary pressure and airway driving pressure increased with CENP possibly indicating increased VILI. However, the tidal movement was possibly constrained at high PEEP values by the shell and additional CENP might have had no additional effect of stabilisation, at a fully recruited lung. Prolonged expiration or a more unstable heterogeneous model of ARDS is needed for a thorough evaluation of this effect.

Mechanical power was only significantly affected by CENP during zero PEEP, while there were no differences during PEEP of 7 and 15 cmH_2_O. The fact that CENP reduced mechanical power during zero PEEP is likely explained by decreased elastance and resistance, while two other variables contributing to MP, e.g. V_T_ and RR, did not differ between CENP levels. During PEEP of 15 cmH_2_O, the significant but small increase of the driving pressure obviously did not result in increased mechanical power, while no other determinants of MP did differ between CENP levels during the highest PEEP.

It is worth noting that the reduction of CO and MPAP depended on the position of the shell, that is, the region CENP was applied, as well as its pressure level. This observation might be explained by differences in venous pooling. These findings contrast with the results of a trial in patients with ARDS showing that locally applied external negative pressure improved cardiac indexes and blood pressure [11]. However, in the latter study, CENP was applied using a poncho-like system wrapping the complete upper thorax and upper abdomen. Similarly, MAP and CO did not differ between PPV with and without CENP, while it decreased over time during conventional PPV without CENP in an iron lung-like cast wrapping pigs from the lower limbs up to the xiphoid level [9]. Additionally, in that study [9], a CENP of – 5 cmH_2_O was applied, which is considerably higher than the pressures used in the present trial. In a bacterial infection model in sheep, CENP ranging between − 60 and – 80 cmH_2_O induced significant haemodynamic impairments [26]. It is worth of note that while a CENP of – 20 cmH_2_O increased TP_caud_, shifted the CoV and increased oxygenation, haemodynamic impairment occurred only at lower CENP levels.

## Limitations

This study has limitations. First, it was explorative in nature, due the lack of pre-existing data, which precluded sample size estimation. However, it suggests that regional, that is, non-whole-body CENP has important effects on respiratory mechanics, distribution of ventilation and oxygenation. Second, the results have been gained using a lavage-only model mimicking only the homogenous loss of surfactant feature of ARDS, which is known for its good recruitability. This limits extrapolation to other ARDS models as well as to the clinical scenario. However, the recruitability of this model was intended, as we sought to investigate the effects of a wide range of applied negative pressures on different PEEP levels. Furthermore, this model allowed significant respiratory impairment but stable haemodynamic conditions. Third, trans-diaphragmatic and intra-abdominal pressures, as well as end-expiratory lung volumes, were not measured, and we were not able to explain the differences between thoracic and abdominal shell position. Fourth, each CENP was applied for a relatively short time, and therewith, the duration of each PEEP level was limited to 15 min. Thus, we cannot exclude that prolonged CENP would have more pronounced effects, especially on haemodynamics. Sixth, conductivity drift between EIT belt electrodes and skin may have led to an overestimation of the effect of CENP on CoA. Seventh, the crossover design of the study precluded the assessment of lung injury. Therefore, we do not know whether the beneficial effects on respiratory function and mechanics are compatible with lung protection.

## Conclusions

In this model of acute respiratory distress syndrome in pigs under PPV, CENP increased the end-expiratory caudal transpulmonary pressure. CENP led to a shift of lung aeration and ventilation towards dependent zones as well as improved respiratory mechanics and oxygenation, especially when applied to the abdomen as compared to the thorax. CENP values ≤ 20 cmH_2_O impaired the haemodynamics.

## Data Availability

The datasets used and/or analysed during the current study are available from the corresponding author on reasonable request.

## Abbreviations

%E_2_: Index of over-distension
∆P_AW_: Driving airway pressure
aBGA: Arterial blood gas analysis
ARDS: Acute respiratory distress syndrome
CENP: Continuous external negative pressure
CO: Cardiac output
CoV: Centre of ventilation
CPAP: Continuous positive airway pressure
E: Elastance
ECG: Electrocardiogram
EIT: Electrical impedance tomography
F_I_O_2_: Inspired fraction of oxygen
I:E: Inspiratory to expiratory time ratio
i.v.: Intravenous
IPPV: Intermittent positive pressure ventilation
M: Molar
MAP: Mean arterial pressure
ME: Mechanical energy
MP: Mechanical power
MPAP: Mean pulmonary arterial pressure
MV: Minute volume ventilation
MW: Mechanical work
NPV: Negative pressure ventilation
PaCO_2_: Arterial partial pressure of CO_2_
PaO_2_: Arterial partial pressure of O_2_
PaO_2_/F_I_O_2_: Fraction of arterial partial pressure of O_2_ and inspired fraction of O_2_ (Horovitz-Index)
P_AW_: Airway pressure
PEEP: Positive end-expiratory pressure
PPV: Positive pressure ventilation
R: Resistance
ROI: Region of interest
RR: Respiratory rate
SD: Standard deviation
TP: Transpulmonary pressure
TP_caud_: Caudal transpulmonary pressure
VATS: Video-assisted thoracoscopy
VILI: Ventilator-induced lung injury
V_T_: Tidal volume
